# Occurrence, Pharmacological Properties, Toxic Effects, and Possibilities of Using Berries from Selected Invasive Plants

**DOI:** 10.3390/antiox14040399

**Published:** 2025-03-27

**Authors:** Simona Oancea

**Affiliations:** Department of Agricultural Sciences and Food Engineering, Lucian Blaga University of Sibiu, 7–9 Dr. Ion Ratiu Street, 550024 Sibiu, Romania; simona.oancea@ulbsibiu.ro; Tel.: +40-2-6923-6441

**Keywords:** invasive alien plants, berries, traditional use, pharmacological activity, antioxidant, toxicity

## Abstract

Invasive alien plants are typically associated with negative impacts on ecosystems and sometimes on health, but studies also describe their ethnomedicinal usage, particularly by indigenous communities. Given the existing limitations regarding a critical study on the berry-producing invasive plants, this study aims to provide scientific evidence and much-needed updated knowledge on the pharmacologically significant fruits of several berry-producing invasive alien plants. A list of 35 species from 16 families is provided, along with their characteristics, historical medicinal uses, updated biological activities, and available toxicity data. The definitions, terminology, and classification criteria used to describe alien species, specifically the invasive types, are also provided. Most of the berries of the described species exhibit remarkable antioxidant properties due to their abundance of highly reactive molecules, mainly polyphenols and carotenoids. Other biological activities, ranging from antimicrobial, anti-inflammatory, and anti-diabetic to anticancer and neuroprotective activities, have been identified. In contrast, quantitative toxicity issues have been poorly studied for berries from invasive plants. Hopefully, this work will serve as a starting point for further exploration of the molecules from berries of these plants in terms of drug discovery to advance various therapies or other applications.

## 1. Introduction

Terrestrial plants, which are of major ecological value for providing food and habitats for many species in a given area, are classified into native and non-native groups, based on their occurrence conditions. While native plants occur naturally in a particular geographic area, the non-native types (also called “alien”) have been introduced, intentionally or accidentally, into areas other than their native regions due to human intervention [1]. Alien plants may become invasive due to the fact that in the invaded area, there is a lack of the specific natural enemies (insects, pathogens) found in the plant’s area of origin, the absence of predators which play an important role in population control, and due to some specific acquired characteristics that are useful for plant competition. Some invasive alien plant species (IAPS) from terrestrial environments can also impact human health, either through direct exposure causing allergic reactions, such as those produced by ragweed (*Ambrosia artemisiifolia*), giant hogweed (*Heracleum mantegazzianum*), and Brazilian pepper-tree (*Schinus terebinthifolius*), or as a vector, as in the case of *Lantana camara*, a plant which affords shelter to the tsetse fly (*Glossina* spp.), which transmits sleeping sickness [2].

From an ecological point of view, since the mid-20th century, the study of invasive alien species has become a very hot topic for several key reasons: (1) globalization, which increased the introduction of non-native species, with immediate consequences for agriculture and biodiversity; (2) economic impacts, in particular those on agriculture, which motivated academics and policy makers to invest in research and management strategies; (3) climate changes, which might favor the spread of non-native species, causing international organizations, e.g., the European and Mediterranean Plant Protection Organization (EPPO), the Center for Agriculture and Bioscience International (CABI), the Intergovernmental Science-Policy Platform on Biodiversity and Ecosystem Services (IPBES), and the Invasive Species Specialist Group (ISSG) of the International Union for Conservation of Nature (IUCN), to become involved in the research and control of invasive alien species by establishing international treaties, e.g., the Convention on Biological Diversity (CBD) and the Global Invasive Species Program (GISP) [3]. To this end, regulations such as the EU Regulation on the Prevention and Management of the Introduction and Spread of Invasive Alien Species, established in 2014 [4], were put in place, with an updated regulation created in 2016 [5]. Collecting data on existing IAPS in specific areas is important for control and management. Thus, a field inventory-based database of alien plants that are present in Romania, both of EU concern and of national interest, was published in 2024 [6]. According to this study, of the 41 plant species of EU concern, 4 are already established and widespread in Romania (*Ailanthus altissima, Asclepias syriaca, Elodea nuttallii, Impatiens glandulifera*), and of the 396 inventoried alien plant species, 7 species were proposed as species of interest for Romania (*Ambrosia artemisiifolia, Ambrosia tenuifolia, Ambrosia trifida, Cyclachaena xanthiifolia, Phytolacca americana, Phytolacca acinose, Verbesina encelioides*).

A huge number of scientific papers continue to be published on IAPS, dealing with their negative impacts on ecology/biodiversity, economy/agriculture, and human health, as well as with strategies of control and prevention, which are deeply related to invasion science, while a much smaller number refer to the positive attributes of such plants. Usually, the beneficial effects of different parts (roots, leaves, fruits, seeds) of IAPS are linked to their ethno-medicinal uses for treating various diseases [2], most of them being linked to the phytotherapy usage by local communities of different geographic areas. Some other positive roles of certain IAPS have been described as follows: (1) phytoremediation by scavenging toxic metals or organic contaminants; (2) sources of bioenergy and animal feed; (3) reduction in soil erosion; (4) attraction of insect pollinators; (5) nitrogen fixation. Due to their accessibility and abundance, IAPS may provide opportunities for conversion into products that are useful for various industries (e.g., pharmaceuticals, textiles, pulp and paper industry, food), one example of such application being the project “APPLAUSE—from harmful to useful with citizens’ led activities”, which focuses on processing the biomass of 25 IAPS into valuable products [7]. Other mentioned applications of IAPS refer to their use as livestock forage, specifically in resource-degraded regions, or as the source of livelihoods in local communities with low incomes [8]. However, evaluation of such benefits should be regarded at a local scale within a certain context, while globally, IAPS often cause long-term environmental damage.

Another facet of the topic is that, due to their chemical composition and historical use as medicinal remedies or food, particularly by indigenous communities, IAPS show high potential for the development of valuable bioactive ingredients for biomedical applications, according to published research [9,10]. This subject will be addressed and documented in the present review paper, especially in the context that currently, the best control strategy for IAPS is based on their removal, which may be achieved mechanically or chemically. Research on invasive plants is becoming even more important since, due to climate changes, we are currently facing the global loss or reduced availability of medicinal plants—a significant source of healthcare products—which can be thus overcome by using alternative plant resources, which have historically been used in some geographical areas. The role of secondary metabolites produced by IAPS and mainly released from roots and leaves has been well documented as a chemical basis of their invasiveness, due to various effects such as allelopathy, anti-herbivore and antimicrobial effects [11]. Several phytochemicals, such as catechins, 8-hydroxyquinoline, terpenes, sesquiterpene lactones, anthraquinones, sugars, protein and other metabolic by-products are involved in the mechanisms of invasion, as described by the authors. The biosynthesis of such compounds in great amounts in different parts of the invasive plants determines the wide range of their biological activities, particularly their strong antioxidant activity. Therefore, invasive plants may become important botanical sources to produce extracts or to isolate bioactive compounds useful for a wide range of purposes.

Numerous IAPS produce fruits with chemical compositions that vary by species, but with some common characteristics that contribute to their invasiveness, such as fewer seeds of medium size (<10 per fruit), which can be dispersed over large areas, and small, attractive fruits with longer fruiting periods that can be easily consumed by birds [12,13]. Some IAPS fruits are dispersed both by birds and mammals (e.g., Rubus *armeniacus* edible berries) [14], while others, e.g., those produced by *L. camara*, are toxic only to mammals, so their limited destruction by herbivores alone supports their high invasiveness [15].

The berries of IAPS have been poorly investigated compared to the other parts of these plants, so the present comprehensive review aims to provide scientific evidence and much-required updated knowledge on the pharmacologically significant fruits of several berry-producing invasive alien plants. A simple search by topic using the database Web of Science/WoS Core Collection (Clarivate Analytics) accessed on 1 March 2025, using the keyword “invasive alien plants” associated with any of the terms “leaves”, “flowers”, “roots”, “fruits”, for the period 1975–2025, led to the following results: 960 documents on leaves, 521 documents on flowers, 504 documents on roots, and 355 documents on fruits. Most of the documents on the fruits of the invasive alien plants were published in the research areas Environmental Sciences Ecology, Plant Sciences and Biodiversity Conservation, and very few in the fields of Life Sciences, Biomedicine, Food Science or Chemistry, probably due to the fact that most of these species are decorative plants. Almost half of the studies indexed in WoS were published in the last eight years, indicating an increasing interest in this topic. Some of the IAPS berries continue to be used medicinally or culinary by local communities, due to their nutrient content (fiber, vitamins, minerals, antioxidants) and due to their historically proven health effects related to valuable chemical compounds, bioactive phytochemicals and pigments (anthocyanins, betalains, carotenoids, lycopene), but more studies are required to help us understand their benefits in relation to their safety. The selected species were described in terms of characteristics and habitats, historical uses, biological activities, and available toxicity data. The results of this approach support the potential valorization of the IAPS extracts or their chemical components for the production of drugs or other useful commodities, as this is an alternative and different way to control invasive plants. A list of 35 berry-producing IAPS is provided and proposed to be further investigated in terms of the mechanism of action of their bio-compounds, and of their toxic effects, which should be evaluated by in vitro and in vivo studies. The information provided in this review paper is presented in a very useful manner, and is organized in user-friendly tables to help readers to easily navigate the data.

## 2. Applied Methods

This study was conducted by collecting qualitative and quantitative literature data using a semi-structured method based on the narrative review type [16]. The methodology was based on searching the following sources of information in English language: (1) worldwide accepted databases of the international organizations dealing with IAPS, which provide information on the distribution data, morphological characters, uses and images: the CABI Compendium Invasive Species, the GISD, the Weed Science Society of America (WSSA) Composite List of Weeds, The EPPO Global Database, the United States Department of Agriculture (USDA) Plants Database, The Dataset/Inventory of Alien Invasive Species in Europe (DAISIE), and The Invasive Plant Atlas of the United States; (2) the Food Plants International (FPI) database, which was particularly useful for extracting information on those parts of the plant species that are edible; (3) research articles (both original articles and reviews); and (4) books. The selection criteria were based on the title and abstract. The study started with the identification of IAPS that produce berries or berry-like fruits; these were identified using online databases, which resulted in the selection of a total 35 berry-producing terrestrial invasive alien plants, followed by applying a keyword search for each specific IAPS using several multidisciplinary scientific databases (SCOPUS, Web of Science), scientific publisher databases (ScienceDirect, SpringerLink, Wiley Online) for relevant full-text original articles, review-type articles, books, and scientific reports. General search terms with different spellings, such as “invasive species terminology”, “invasive alien plant species”, “terrestrial invasive alien plants”, “list of invasive alien plants”, “biological activity of invasive alien plant species”, “toxicity of invasive alien plant species”, “application of invasive alien plant species”, and specific search terms were used. The strategy for specific search terms was based on a keyword consisting of the scientific name of each selected plant (e.g., Viburnum *opulus*) OR its common names, and combinations using the Boolean operators: “scientific name” AND “traditional use”, “scientific name” AND “medicinal use”, “scientific name” AND “ethnopharmacological use”; “scientific name” AND “fruits” OR “berries”; “scientific name” AND “biological activity” OR “pharmacological activity” OR “toxicity”. For the latter strategy, which was applied to describe the pharmacological properties and toxicity of berry-producing invasive plants, articles were removed if they were not concerned with the bioactivity or properties strictly related to the fruits of the species.

The results were integrated into the tables as much as possible. Moreover, a comprehensive classification of plants used in studies on invasive plants was described on the basis of nine eligible frequently used criteria: occurrence, residence time, means of introduction, type of encountered habitat, invasion status, impact, and the Environmental Impact Classification for Alien Taxa (EICAT)-based scoring of the environmental impact.

The paper was structured in three main sections, as follows: (1) definitions, terminology and classification of plant species; (2) characteristics and historical uses of 35 berry-producing invasive alien plants; (3) current knowledge on the pharmacological properties and toxicity of selected IAPS’ berries. Representative images illustrating the fruits of the selected IAPS were included by mentioning the photo sources from the globally accepted databases mentioned.

## 3. Definitions, Terminology, and Classification of Plant Species

Plant species that populate a given geographic area are primarily classified into the indigenous (native) group when they occur naturally, and the alien (non-native) group when they do not occur naturally in the specified geographic location. Non-native species are introduced to an area not previously populated by said species, as a result of direct or indirect human activities. This does not mean that these species are necessarily harmful to that environment or economy. When such species develop the potential to produce self-sustaining populations in the new area without human aid, they are defined as naturalized. Naturalized species that threaten or have a detrimental effect on biodiversity and related ecosystem services, producing environmental, economic, or human harm are called invasive species.

There is a general debate regarding the numerous terms and classification criteria used by scientists to describe alien species, particularly the invasive types, which may create confusion, incorrect use, and limited understanding. Alien species have been classified according to different criteria: time of appearance, pathway, habitat, degree of naturalization, and impacts. For a historical view of definitions and interpretations of the term “invasive” when applied to plant species, readers are invited to consult the paper of Pyšek [17].

The most globally accepted and frequently encountered criteria of classification of plant species are described in Table 1, based on the information covered by the works of Pyšek [18,19] and Mitić [20]. The criteria for alien plants are as follows: residence time, means of introduction, type of encountered habitat, invasion status, impact, EICAT scoring of impact, purpose, and vector of plant introduction. According to an updated review regarding the European alien flora [21], archaeophytes represent 1.5% and neophytes represent 77.2%; the remaining percentage was attributed to archaeophytes/neophytes in relation to the region, and to undistinguished alien status. Based on the same work, a classification of European alien plants according to their invasion status showed that 33.3% of aliens are casual, 36.3% are naturalized, and 14.4% are invasive in at least one European area. The magnitude of the environmental impact of IAPS can be evaluated according to the EICAT scheme, with alien plants being classified into five impact categories, from minimal concern to massive concern [22]. Species that fall into the categories moderate, major, or massive are considered harmful. EICAT is a useful tool for scientists, conservation practitioners, and policy makers, because it helps to inform the prioritization of invasive alien species of great concern in a given area.

A standardized list of seven terms to be used for invasive species (which is particularly useful for stakeholders) has been introduced, with the purpose of delivering a simplified harmonized message associated with the impacts of invasive species [23]: “native”, “non-native”, “introduced”, “established”, “invasive,” “nuisance” and “range change”. The authors considered that the terms “native invasive”, “invasive exotic”, “invasive weed”, “alien”, “foreign” and “non-indigenous” should be avoided due to confusion and misinterpretation.

The educational issues regarding the correct usage of terms and accurate identification of plants as invasive are essential for preventing the introduction and spread of new invasive plants. It has been estimated that the number of new invasive plants has continued to multiply over the years [24] mainly due to an increased mobility of people and globalization of economies [23].

## 4. Characteristics and Historical Uses of 35 Berry-Producing Invasive Alien Plants

It is considered that at least 4% of all known vascular plant species have become established alien plant species [25] which grow outside their natural area due to human assistance; at present, this amounts to a total of 13,939 species [26], but this number is continuously increasing. Most of the naturalized alien species are concentrated in North America and Europe. According to the most recent data, 2981 invasive plant taxa have been recorded [27].

Within the European and Mediterranean region, according to the updated information published by the EPPO database [28], 51 plant species are found on the “List of invasive alien species”, 25 species are on the “Observation List of invasive alien plants”, while 15 IAPS are on the “Alert List”. In the EU, the EU Regulation 2014 [4] set requirements on the adopted “List of invasive alien species of Union concern” to be updated as needed; the 2019 updated list now includes a total number of 41 IAPS [29].

The present work focuses on IAPS that produce berry-like fruits, which abound in valuable chemical compounds of pharmacological interest, which may turn such “enemies” into sources of bioactive products. Thirty-five species of berry-producing were selected according to the inventory of IAPS, either in Europe (EPPO, DAISIE) or globally (ISC Invasive Species Compendium by CABI, WSSA Composite List of Weeds 2023 Update). Most berry-producing IAPS are woody plants, perennial shrubs, or trees with small, beautifully colored fleshy fruits, which may be singular or in clusters, and are attractive for frugivore birds, which not only eat them but also contribute to their spread and invasiveness. Some of these species produce edible berries and/or berry-like fruits, such as *Berberis thunbergii* [30], *Elaeagnus umbellata* [31], *Solanum sisymbriifolium* [32], but most of their fruits were reported to be non-edible or toxic. However, such fruits may constitute a valuable source for producing drugs or preparing extracts and products rich in biologically active chemical compounds. For this reason, studying the composition and biological/pharmacological effects of the fruits of IAPS may be viable method for controlling invasiveness through consumption, as harvesting the fruits may limit the spread. Although many IAPS exhibited substantial effects on the invaded ecosystems, Shackleton et al. [33] indicated that 16% of invasive alien species can be categorized as desirable and weakly competitive due to various benefits they offer, in particular to human livelihood. The work of Nuñez et al. [34] describes some benefits of using invasive species for food that increase the awareness of invasive species, help the non-scientific community to identify invasive species, or boost the local economy, but also draw attention to some risks, particularly those related to the creation and maintenance of a market of such products which may lead to the protection of some harmful species.

With regard to the berry-producing IAPS selected in the present work, their scientific and common names, species description and knowledge about their invasiveness, habitats, and historical uses of different parts of the plants in traditional medicine or as food are summarized in Table 2. The parts of the plants that are edible are described according to the information within the cited paper or the FPI database [35]. IAPS producing berries or berry-like fruits were grouped according to the families to which they belong, as follows: *Adoxaceae, Aquifoliaceae, Anacardiaceae, Araliaceae, Asparagaceae, Berberidaceae, Caprifoliaceae, Celastraceae, Cornaceae, Elaeagnaceae, Oleaceae, Phytolaccaceae, Rosaceae, Solanaceae, Verbenaceae*, and *Vitaceae*.

**Table 2:** List and description of 35 berry-producing terrestrial invasive alien plant species.

Scientific Name		Common Name(s)	General Species Description	Range/Invasiveness/Coverage	Habitat	Historical Uses (Medicinal, Food, Dying)	Ref.
Family	Genus and Species						
Adoxaceae (formerly Caprifoliaceae)	*Viburnum opulus*	Guelder Rose, European or American Highbush Cranberry, Cranberry Bush, Crampbark, Snowball Tree, Whitten Tree, Summer Berry	Perennial shrub or tree, growing up to 38 cm (shrub) or 4 m (tree), simple leaves, white flowers, reddish-orange fruits.	Native to Europe, Caucasus, Siberia, and Central Asia. Introduced to the UK, China, Canada, and USA. Invasiveness category 2 in the upper Midwest (lesser invader of natural areas).	Open woods, forest edges, hedges, thickets, garden bog, and damp soils.	Folk medicine: cardiotonic, bark used for muscular pains, homeopathy, antispasmodic; leaves and fruits used as antiscorbutic, laxatives, and emetics. Food use: flowers (tea); raw or cooked fruits (jelly).	[36,37,38,39,40,41,42]
Aquifoliaceae	*Ilex aquifolium*	Holly, Christmas Holly, Christ’s Thorn, English Holly, Common Holly, Holly Green	Perennial shrub, tree growing up to 20 m, evergreen leathery leaves, white flowers, red to orange fruits.	Native to Europe, Northern Africa, and Asia. Introduced and naturalized within the USA, Canada, Australia, New Zealand, and India. WSSA Composite List of Weeds.	Shaded or sunny areas; forests.	Herbal medicine: roots used to treat cough, gravel, and tuberculosis; leaves used to treat influenza, malaria, bronchitis, and rheumatic complaints; fruits used as emetics and purgatives. Food use: leaves (tea); fruit (drink).	[37,39,41,42,43,44]
Anacardiaceae	*Rhus typhina*	Staghorn Sumac, Velvet Sumac, Indian Lemonade	Perennial shrub, tree, growing up to 9 m, lance-shaped leaves, yellowish green to white flowers, red fruits produced in clusters.	Native to USA. Introduced to Europe, Central Asia. CABI Invasive Species Compendium (ISC). WSSA Composite List of Weeds.	Forest edges, prairies, disturbed environments, old fields, fencerows, and roadsides.	Herbal medicine (North American Indian tribes): bark used as antirheumatic, anthelmintic, tonic, emetic, analgesic, stomachic, blood purifier, for sunburn blisters, boils, colds, coughs, sore throats, mouth sores, tuberculosis, fever, stomach pain, diarrhea, rheumatism, and venereal disease; leaves for asthma, diarrhea; fruits used for gastrointestinal disorders. Food use: leaves; fruits (refreshing lemonade-like drink called “sumacade” or “Indian lemonade”); fruit powder used as spice.	[36,42,43,44,45,46,47]
	*Schinus terebinthifolia*	Brazilian Pepper Tree, Pepper Berry, Pink Pepper, Christmas Berry, Schinus	Perennial evergreen shrub, tree growing up to 9–10 m, alternate dark green leaves with pepper or turpentine odor when crushed, white to yellow flowers, small red fruits.	Native to Brazil, Argentina, Paraguay, and Uruguay. Introduced to Europe, Central America, and Australia. CABI Invasive Species Compendium (ISC). WSSA Composite List of Weeds.	Natural and disturbed areas, forest borders, river margins, old fields, and wetlands.	Traditional Brazilian medicine: anti-inflammatory, used to treat respiratory diseases. Food use: spice (South and Central America), drink from dried fruits (South America), alcoholic drink from fruits (natives of the Andes); sometimes found as an adulterant of black pepper.	[44,48,49]
Araliaceae	*Hedera helix*	English Ivy, Common Ivy	Perennial vine, evergreen simple leaves, yellow small flowers, dark blue to black fruits.	Native to Europe, Western Asia, and Northern Africa. Introduced to the USA, Australia, Canada, India, and New Zealand. CABI Invasive Species Compendium (ISC); WSSA Composite List of Weeds. Invasiveness category 2 in the upper Midwest (lesser invader of natural areas).	Woodlands, forest edges, rocks and cliffs, gardens, hedges, urban areas, and alkaline soils.	Folk medicine: leaves and flowers used as an antimicrobial, antipyretic, expectorant, antispasmodic; leaves and berries used for the treatment of cough and bronchitis. Homeopathy: treatment of coughs, colds, emphysema, and asthma. Pink and gray dye for wool and textiles	[36,37,39,41,43,44,50,51,52]
Asparagaceae	*Asparagus asparagoides*	Bridal Creeper, African Asparagus Fern	Perennial climbing herb, solitary and alternate leaves, greenish white scented flowers, and red globular fruits.	Native to South Africa. Introduced to Australia, New Zealand, the USA, and Europe. CABI Invasive Species Compendium (ISC); EPPO Observation List of invasive alien plants.	Woodlands, forests, coastal areas, and riverbanks.	South African traditional medicine: roots used to treat bathe sore eyes; tubers used as a remedy for stomach disorders.	[28,41,43,53]
Berberidaceae	*Berberis thunbergii*	Japanese Barberry, Thunberg’s Barberry	Deciduous shrub growing up to 2.4 m, simple alternate leaves, yellow flowers, small red fruits.	Native to Japan. Introduced to Central Europe, Central Asia, China, Korea, and North America. WSSA Composite List of Weeds. Invasiveness category 2 in the upper Midwest (lesser invader of natural areas).	Woodland edges, open woods, wetlands, roadsides, fence rows, old fields.	Traditional medicine: roots used as an antihelminthic, antibacterial, antiseptic, for healing wounds; fruits used to purify blood, antimicrobial (Ayurvedic medicine) properties. Food uses: fruits used in Persian and Georgian cuisine.	[36,43,44,54]
	*Nandina domestica*	Sacred Bamboo, Heavenly Bamboo, Nandina	Perennial evergreen shrub growing up to 2 m, large alternate leaves, pinkish-white flowers, red fruits.	Native to Japan and India. Introduced to Europe, USA, Australia, and South Africa. Recognized as invasive categories 1 and 2 (USA). CABI Invasive Species Compendium (ISC); WSSA Composite List of Weeds.	Forests, edges, roadsides, and thickets.	Herbal medicine (China, Japan): used to treat a cough and breathing difficulties, such as asthma. Food use: fruits and leaves (edible). Symbol of good luck.	[39,41,42,43,44,55,56]
Caprifoliaceae	*Sambucus ebulus*	Dwarf Elder, Dwarf Elderberry, Danewort, Dane Weed, European Dwarf Elder, Elderwort	Perennial forb/herb growing up to 0.2 m, white flowers, and black fruits.	Native to Europe, Asia. Introduced to USA and the UK. Is considered an overwhelming species in meadow habitats of Romania.	Waste ground, woods, hedgerows, and scrub.	Herbal medicine: roots and leaves used as a laxative, diuretic; root tea used as a remedy for dropsy. Food use: flowers (tea); cooked fruits used for flavoring soups. Natural blue textile dye from fruits. Natural black hair dye from roots.	[37,41,42,43,57]
Celastraceae	*Celastrus orbiculatus*	Bittersweet, Oriental/Asian Bittersweet, Climbing Spindleberry	Woody perennial vine growing up to 10 cm, rounded leaves, small greenish-yellow flowers, green to yellow-orange fruits.	Native to Eastern China, Japan, Korea, and Mongolia. Introduced to Europe, the USA, Canada, and New Zealand. CABI Invasive Species Compendium (ISC); WSSA Composite List of Weeds. EPPO A2 list of pests recommended for regulation as a quarantine pest. Invasiveness category 1 in the upper Midwest (major invader of natural areas).	Forest edges, grasslands, savannas, thickets, and roadsides.	Traditional Chinese and Indian medicine: roots, stems and leaves used as antirheumatic, depurative and tonic; fruits used as an anti-inflammatory, antitumor properties. *The Great Dictionary of Chinese Medicine*: herb used to treat rheumatoid arthritis, injuries from falls, pain in the waist and lower extremities, and amenorrhea. Food use: leaves are considered edible when cooked.	[36,41,42,44,58,59]
	*Euonymus alatus*	Burning Bush, Winged Euonymus, Winged Burning Bush, Winged Spindle Tree	Perennial deciduous shrub growing up to 4.6–6.1 m, elliptical leaves, greenish-yellow flowers, and purple red fruits.	Native to Asia (China, Japan, Korea), Siberia, and Russian Far East. Introduced to the USA, Canada, and Europe. WSSA Composite List of Weeds. Invasiveness category 2 in the upper Midwest (lesser invader of natural areas).	Open woods, forests, hedges, prairies	Traditional Chinese medicine: used for urticaria, dysmenorrhea, wound, dysentery, rheumatism and arthritis; stem used in Korea to treat intestinal worms and cancer. Food use (with caution): leaves and fruits (tea).	[36,41,42,44,60,61]
Cornaceae	*Cornus sericea* *(Cornus stolonifera)*	Red Osier Dogwood, Red Twig Dogwood	Perennial deciduous shrub or tree, ovate to elliptic leaves, white flowers, and white fruits (berrylike drupes).	Native to North America, Mexico. Introduced to Europe. CABI Invasive Species Compendium (ISC); EPPO List of invasive alien plants.	Shores, thickets, wetlands, roadsides, gardens.	Folk medicine: used by native North American Indians to treat diarrhea, fever, skin problems. Food use: fruits (raw or cooked), seeds (edible oil).	[28,36,42,60,62]
Elaeagnaceae	*Elaeagnus umbellata*	Autumn Elaeagnus, Autumn Berry, Autumn Olive, Japanese Silverberry	Perennial deciduous shrub growing up to 3.5–5 m, silvery lanceolate and alternate leaves, white to light yellow flowers, and coral pink fruits.	Native to Asia (China, Japan, Korea). Introduced to Europe, the USA, Canada, and India. CABI Invasive Species Compendium (ISC); WSSA Composite List of Weeds. Invasiveness category 1 in the upper Midwest (major invader of natural areas).	Sparse woods, thickets, forest edges, prairies, grasslands, disturbed environments, and roadsides.	Traditional Asian medicine: roots used as an antirheumatic, astringent, or for the treatment of cough; seed oil used for pulmonary diseases; flowers used for dysentery. Food use: raw and cooked fruits (jams), seeds (raw or cooked).	[36,41,42,44,63,64]
Oleaceae	*Ligustrum obtusifolium*	Privet, Border Privet	Deciduous or semi-evergreen shrub, opposite simple oval or oblong leaves, white flowers, and purple to black fruits.	Native to Asia (China, Japan, Korea). Introduced to the USA, Canada, and Europe. CABI Invasive Species Compendium (ISC); WSSA Composite List of Weeds.	Woodland, forest edges, roadways, old fields, and disturbed areas.	Traditional Chinese medicine: fruits are tonic; hepatoprotective. Food use: *Ligustrum* spp. leaves (Ku-Ding-Cha) may be used as a tea-like beverage (prevent hypertension, sore throats, inflammation, diabetes); nutritional supplement.	[44,65,66]
Phytolaccaceae	*Phytolacca americana*	Pokeweed, American/Virginian Pokeweed, Pokeroot, Pokeberry, Pigeon Berry, Inkberry, Red-ink plant	Perennial herb, bush growing up to 2.2 m (even 4 m), reddish-purple stems, simple leaves alternate, white flowers, and black fruits.	Native to North America and East Asia. Introduced to Europe, Japan, Australia, and South Africa. Listed as a Class I malignant invasive plant in the Invasive Species List of Yunnan Province (2019 Edition); WSSA Composite List of Weeds. List of invasive alien species of priority for Romania.	Woods, roadsides, disturbed areas, thickets, and pastures. Adapted to various soils; rapid seed spread rate.	Traditional medicine: purgative herb in India, roots used as laxative; used as a treatment for arthritis; leaves used to treat boils. Homeopathic use: fruit tincture used in France for homeopathic treatments of pharyngitis, tonsillitis, and laryngitis. Food use (with caution): fruits (cooked, in pies) and young leaves (cooked) are considered edible parts; fruit pigment used for wine coloration (up to XIX century). Natural dye for wool and textiles.	[37,38,42,43,44,67]
Rosaceae	*Cotoneaster franchetii*	Orange Cotoneaster	Perennial small tree or shrub growing up to 1–3 m, semi-deciduous, oval leaves, pink flowers, rounded and fleshy orange-red, and pinkish-orange fruits.	Native to Indochina and China. Introduced/naturalized within the USA and Europe. CABI Invasive Species Compendium (ISC); WSSA Composite List of Weeds. Listed as a weed in Australia.	Forests, open woodland, and roadsides.	Traditional medicine (*Cotoneaester* spp.): cardiovascular disorders, diabetes, fever, and cough. Food use: fruits.	[41,42,44,60,68]
	*Cotoneaster horizontalis*	Cotoneaster, Rock Cotoneaster, Wall Cotoneaster, Wall-spray	Perennial deciduous or semi-evergreen shrub, usually growing up to 50 cm (sometimes up to 1 m), white flowers, bright red fruits.	Native to China. Introduced to Europe and North America CABI Invasive Species Compendium (ISC).	Thickets, rocks, rocky slopes and dry mountain areas, and urban areas.	Traditional medicine (*Cotoneaester* spp.): cardiovascular disorders, diabetes, fever, and cough.	[41,69,70]
	*Cotoneaster lacteus*	Late Cotoneaster, Milkflower Cotoneaster, Red Clusterberry	Perennial shrub growing up to 2 m, oval dark-green leaves, white flowers in clusters, and red fruits.	Native to China. Introduced/naturalized within the USA and Europe. WSSA Composite List of Weeds.	Gardens, woods, slopes, and grassland.	Traditional medicine (*Cotoneaester* spp.): cardiovascular disorders, diabetes, fever, and cough.	[41,44,71]
	*Cotoneaster pannosus*	Silverleaf Cotoneaster	Perennial shrub growing up to 3 m, semi-evergreen, green oval-shaped leaves, and globose dark red fruits.	Native to China. Introduced/naturalized within Europe, North America, Australia, and South Africa. CABI Invasive Species Compendium (ISC); WSSA Composite List of Weeds.	Thickets, slopes, rocky places, and garden.	Traditional medicine (*Cotoneaester* spp.): cardiovascular disorders, diabetes, fever, and cough.	[41,44,72]
	*Prunus serotina*	Black Cherry, Rum Cherry, Whisky Cherry, Wild Cherry	Perennial deciduous shrub and, on rare occasions, a tree growing up to 20 m. Oblong-ovate leaves, white fragrant flowers, and purple black fruits.	Native to North America. Introduced to Central Europe. CABI Invasive Species Compendium (ISC); EPPO List of Invasive Alien Plants; WSSA Composite List of Weeds.	Forests, woodland, open vegetation, and urban areas.	Traditional medicine (*Cotoneaester* spp.): cardiovascular disorders, diabetes, fever, and cough. Food use: raw and cooked fruits (pies, jellies); seeds (raw or cooked) used in the Mexican diet.	[28,42,43,73,74,75]
	*Rubus armeniacus* *(Rubus bifrons)*	Armenian Blackberry, Himalayan Blackberry	Perennial woody shrub growing up to 3 m, dark green toothed leaves, white to rose flowers, and black fruits.	Native to Armenia. Introduced in North America, South Africa, and Europe. WSSA Composite List of Weeds.	Wetlands, disturbed areas (railway lines, roadsides, fence lines), and recently burnt sites.	Food use: fruits are edible, may be used fresh, frozen, or canned.	[14,44]
Solanaceae	*Solanum carolinense*	Horsenettle, Ball Nightshade, Wild Tomato, Devil’s Potato	Perennial herb growing up to 1.2 m, alternate simple and ovate leaves, violet to white flowers, globular yellow and orange fruits.	Native to Canada and USA. Introduced in Russia, Japan, and Europe. EPPO List A2 of pests recommended for regulation as quarantine pests.	Grain and vegetable fields, roadsides, waste areas, and gardens.	No identified traditional uses. No identified uses in food.	[28,43,76,77,78]
	*Solanum dulcamara*	Bittersweet Nightshade, Deadly Nightshade, Woody nightshade, Climbing Nightshade	Perennial vine, forb, subshrub growing up to 10 m, leaves variable in shapes, purple, violet or white flowers, and red fruits.	Native to Canada. Introduced in Europe, Australia, India, New Zealand, and the Russian Federation. CABI Invasive Species Compendium (ISC); WSSA Composite List of Weeds. Invasiveness category 2 in the upper Midwest (lesser invader of natural areas).	Forest	Herbal medicine: stems and berries used to treat rheumatism, arthritis, and skin disease.	[28,36,37,43,44,79,80]
	*Solanum elaeagnifolium*	Silverleaf Nightshade, Prairie-berry, Silver Bitter-apple, Tomato Weed	Perennial forb/herb, subshrub, simple or shallowly lobed leaves, purple to pale violet or white flowers, and yellow to orange fruits.	Native to Southern USA, North-East Mexico. Introduced/naturalized to Europe, Australia, South Africa, and Japan. CABI Invasive Species Compendium (ISC); Listed in EPPO A2 Lists of invasive alien plants recommended for regulation as quarantine pests. WSSA Composite List of Weeds.	Dry habitats, woodlands, crop lands, and gardens.	Traditional medicine (Kiowas American Indians tribe): seeds used for tanning hides. Food use: cheese making (seeds and berries used by the Pima American Indians tribe).	[28,42,44,81]
	*Solanum mauritianum*	Tobacco Bush/Tree, Earleaf Nightshade, Bug Weed, Woolly Nightshade	Perennial shrub or small tree growing up to 10 m, large elliptic gray-green leaves, mauve flowers, and green to yellow round fruits.	Native to Brazil, Paraguay, Uruguay, Peru, Argentina. Introduced to USA, Australia, India, South Africa, and Europe. CABI Invasive Species Compendium (ISC); WSSA Composite List of Weeds. Noxious weed in several countries (category 1 in South Africa stipulated under the Conservation of Agricultural Resources Act, Class B noxious weed in New Zealand).	Natural and disturbed wet forests, urban areas, roadsides, pastoral land, and waste ground.	Traditional medicine (South Africa): roots used for treating gonorrhea, toothache, excessive menstrual bleeding, wounds; leaves used to treat headache, wounds; macerated fruits used for cleaning kidneys. Food use (with caution): fruits.	[41,42,43,44,67,82,83,84]
	*Solanum nigrum*	Black Nightshade, Blackberry Nightshade	Annual/biennial forb/herb, subshrub growing up to 1 m, ovate leaves, small white, yellow-green flowers in umbels, and purple-black or green to yellowish green globular fruits.	Native to Europe, Asia; Introduced to US, Canada, Japan, and New Zealand. CABI Invasive Species Compendium (ISC); WSSA Composite List of Weeds.	Open forests, wooded savannahs, thicket, cropland, fallow and disturbed sites, and roadsides.	Traditional medicine (Ayurveda, South Africa): roots used as tonic, to treat wounds; leaves used for stomachache, liver disorders, rheumatic joints, laxative, wounds; berries used as laxative, diuretic, aphrodisiac, jaundices, diarrhea, and fever. Food use: leaves, flowers, and cooked fruits (jams, pies).	[41,42,43,44,67,85,86]
	*Solanum pseudocapsicum*	Jerusalem Cherry, Winter Cherry, Star Capsicum, Mirchala	Perennial shrub/subshrub, simple, alternate, elliptic to lanceolate leaves, white flowers, and bright orange-red fruits.	Native to Brazil and Northern South America. Introduced to Europe, Asia, Australia, New Zealand, South Africa, and the USA. WSSA Composite List of Weeds.	Disturbed forests, shady open habitats.	Traditional medicine: leaves used for skin eruptions. Food use: fruits (coloring).	[41,42,43,44,87]
	*Solanum seaforthianum*	Brazilian Nightshade, Italian Jasmine, Potato Creeper, Potato vine	Perennial vine, pinnate leaves, violet or pale violet flowers, and bright shiny red fruits.	Native to South America. Introduced to the USA, Asia, Central and Southern Africa, Australia, and India. CABI Invasive Species Compendium (ISC).	Forests, crops, pastures, grasslands, disturbed environments, and waste areas.	Traditional medicine: treatment of digestive disorders, fever, inflammation, and headache. Food use: fruits.	[41,42,79,88]
	*Solanum sisymbriifolium*	Sticky Nightshade, Wild Tomato	Annual or perennial herb/shrub, simple or pinnate leaves, purplish or white flowers, and bright red fruits.	Native to South America. Introduced to Africa, Asia, Europe, Australia, New Zealand, and the USA. CABI Invasive Species Compendium (ISC); EPPO Observation List of invasive alien plants.	Roadsides, waste areas, landfills, woodlands, and gardens.	Traditional medicine: used as febrifuge, as an analgesic, and used to treat syphilis, hypertension, diarrhea, and respiratory and urinary tract infections. Food use: fruits.	[28,41,42,60,89,90]
	*Solanum viarum*	Tropical Soda Apple	Perennial subshrub growing up to 1.5 m, ovate dark-green leaves, white or yellowish flowers, and yellow globose fruits.	Native to Argentina, southern Brazil, Paraguay, and Uruguay. Introduced to the USA, Mexico, India, South Africa, Australia, and Europe. EPPO Observation List of invasive alien plants; WSSA Composite List of Weeds.	Grasslands, thickets, roadsides, river banks, agricultural field, and pastures.	Indian traditional medicine: roots and leaves used for dysentery; fruits used to treat wounds and toothache. Food use: fruits.	[28,41,42,43,44,67,79,91]
	*Cestrum nocturnum*	Queen of the night, Night Cestrum, Night Jasmine	Perennial glabrous shrub growing up to 5 m, ovate-oblong, petiolate and obtuse leaves, greenish-white or pale greenish-yellow flowers exhibiting strong sweet fragrance at night, and white fruits.	Native to South America. Introduced to the USA, Australia, India, Japan, and New Zealand. CABI Invasive Species Compendium (ISC).	Wet forests, thickets, disturbed environments, and gardens.	Indian traditional medicine: stem bark and leaves used as sedative; antimicrobial properties; flowers used as cardiotonic.	[41,79,92]
	*Lycium ferocissimum*	African Boxthorn	Perennial woody shrub or tree growing up to 5 m, obovate to oblong bright green leaves, white to mauve scented flowers, and orange to red globular berries	Native to South Africa. Introduced to Australia, New Zealand, and Cyprus. EPPO List of invasive alien plants. WSSA Composite List of Weeds. Regarded as a Weed of National Significance in Australia.	Coastal areas, waterways, roadsides, and waste areas.	Traditional medicine: detoxification in case of narcotic poisoning. Food use (with caution): fruits; honey from flowers.	[28,42,44,93,94,95]
Verbenaceae	*Lantana camara*	Lantana, Common Lantana, Bunch Berry, Cherry Pie, Shrub Verbena, Tuck-berry, Wild Sage; Banana Tea	Perennial evergreen shrub growing up to 4.6 m; ovate leaves with unpleasant odor when crushed; orange, pink, or white flowers; and small green to black fruits.	Native to Central and South America. Introduced to Europe, India, the USA, and Australia. WSSA Composite List of Weeds.	Woodlands, gardens, and cultivated lands.	Traditional medicine (Ayurveda): decoction from roots used for tetanus, rheumatism, and malaria; leaves are febrifuge, used for cold and for healing wounds.	[43,44]
Vitaceae	*Ampelopsis glandulosa (var. brevipedunculata, heterophylla)*	Porcelain Berry, Creeper, Wild Grape, Turquoise-berry Vine, Amur Peppervine	Perennial deciduous vine growing up to 6 m; heart-shaped leaves; small, greenish-white flowers, and blue, purple, and green fruits.	Native to China, Japan, Korea, and Russia. Introduced to Canada and the USA. WSSA Composite List of Weeds. Invasiveness category 3a in the upper Midwest (likely to become a major invader).	Forest edges, grasslands, riverbanks, open areas of the urban landscape, waste sites, and roadsides.	Chinese traditional medicine: stem and roots used as an anti-inflammatory, diuretic, and anti-hepatotoxic; berries used as depurative, hepatoprotective, and febrifuge. Food use: leaves (cooked) and fruits (raw or cooked).	[36,42,43,44,96]
	*Parthenocissus quinquefolia*	Virginia-creeper, American Ivy	Perennial vine, alternate leaves palmately divided; small white or green flowers; and dark blue fruits in a terminal cluster.	Native to the Eastern USA, South-Eastern Canada, and Mexico. Introduced to Europe. CABI Invasive Species Compendium (ISC); WSSA Composite List of Weeds.	Forests, woodland edges, oak-hickory and bottomland forests, thickets, and roadsides.	Folk medicine (Native Americans): roots used to treat gonorrhea and diarrhea; bark used as a tonic, expectorant, and remedy for dropsy; leaves used as astringent and diuretic; fruits used as febrifuge. Food use: fruits (raw), roots, and stems (cooked).	[36,40,42,43,44,60,97]

According to the information summarized and referenced in Table 2, while most fruits of IAPS have been historically and mainly used as medicinal remedies in different cultures, the berries of several IAPS continue to be traditionally consumed as food, either in their fresh state (*V. opulus, B. thunbergii, N. domestica, P. serotina, A. glandulosa, P. quinquefolia*), or in a processed form, e.g., cooked (*S. ebulus, C. stolonifera, P. americana, P. serotina, S. elaeagnifolium, S. nigrum, A. glandulosa*), or in the form of drinks (*I. aquifolium, R. typhina, S. terebinthifolia, E. alatus*), jellies (*V. opulus*) or dried spices (*R. typhina*). These documented issues may lead people to consider that these fruits are edible and will not have side effects on human health. For this reason, further scientific evidence of their benefits and potential toxic effects is provided in the next section of this paper.

## 5. Current Knowledge on Pharmacological Properties and Toxicity of Selected IAPS’ Berries

In this section, the pharmaceutical value of the 35 selected berry-producing IAPS with historical medicinal use has been explored, as well as the toxicity of their fruits. The scientific literature indicates a range of biological activities of fruits of IAPS, often influenced by their chemical composition, a fact that gives them applicative potential in the medicine, cosmetic, food and/or textile industries.

An updated and constructive analysis unveiling the bioactive potential of fruits of the 35 studied berry-producing IAPS from 16 families is summarized in Table 3, which describes the fruits’ characteristics, their pharmacological activities, and their toxicity. The research published so far shows a wide range of biological activities of IAPS berries and berry extracts, e.g., antioxidant, anti-inflammatory, neuroprotective, anticancer, anti-diabetic, antimicrobial, hepatoprotective, antihypertensive, antidiarrheal, antiemetic, etc.

Due to the high amount of specific secondary metabolites known to quench harmful biological radicals, primarily phenolic compounds, but also carotenoids, alkaloids, and terpenes, berries from most IAPS (porcelainberry-*A. glandulosa var. brevipedunculata*, Japanese barberry-*B. thunbergii*, bittersweet-*C. orbiculatus*, red osier dogwood-*C. stolonifera*, autumn olive-*E. umbellata*, African boxthorn-*L. ferocissimum*, sacred bamboo-*N. domestica*, Virginia-creeper-*P. quinquefolia*, pokeweed-*P. americana*, Chinese-sumac-*R. typhina*, dwarf elderberry-*S. ebulus*, Brazilian pepper-*S. terebinthifolius*, *Cotoneaster* spp., and *Solanum* spp.) exhibited strong antioxidant activity [49,58,63,94,96,98,99,100,101,102,103,104,105,106]. The ethanol extract of *A. brevipedunculata* berries showed the capacity of decreasing hepatocyte injury through the decrease in lipid peroxidation, particularly in the presence of Fe(II) [96]. Studies on the fruits of several *Cotoneaster* spp, including *C. horizontalis* and *C. pannosus*, have demonstrated strong antioxidant activity within their ethanol extracts in a concentration-dependent way, which is comparable to or greater than that of synthetic antioxidants (BHA, BHT), by using chemical models based on in vitro assays (FRAP, DPPH), but also by using more physiologically relevant assays, such as linoleic peroxidation-TBARS [70]. The strong antioxidant activity was attributed to polyphenols. The methanol extracts obtained from fruits of *E. umbellata* showed antioxidant activities in a dose-dependent way, measured by DPPH and FRAP assays [63]. The ethanol extracts obtained from unripe berries of *L. ferocissimum* demonstrated higher DPPH- and ABTS^+^-radical scavenging activities than those found in extracts obtained from ripe fruits or from leaves [94]. The authors showed that the iron (II)-chelating activity of extracts from unripe fruits was comparable to that of the commercial antioxidant BHT.

The study by Marinas et al. [103] showed that the antioxidant activities as measured by TEAC and DPPH assays of the ethanol extract of pokeweed berries (*P. americana*) were lower than those of the leaf extracts. Extracts from lyophilized berries of *P. serotina* exhibited antioxidant activities (DPPH, FRAP) higher than those obtained from edible berries, such as plums and grapes [74]. Because optimization of extraction conditions may enhance the biological properties of extracts, our research group investigated different process parameters in relation to the antioxidant activity of *R. typhina* dried fruits, showing that a solvent/solid ratio of 20/1 (v/w), 61.51% aqueous ethanol, and 10 min extraction time using an ultrasound-assisted technique, are optimal (Box–Behnken design) for efficient bioactive content and antioxidant activity (FRAP, DPPH) [107]. The study of Kossah et al. [104] showed that the IC_50_ value for the antioxidant activity by DPPH of the ethanol extract obtained from ripe dried fruits of *R. typhina* was lower (0.016 mg/mL) than that of ascorbic acid used as control (0.019 mg/mL), while the opposite was found with regard to the reducing power of the samples. The DPPH-IC_50_ value of the *S. elaeagnifolium* fruit extract (71.21 ± 3.87 μg/mL) was found to be comparable to that of the standard (BHT, IC_50_ = 67.17 ± 2.04 μg/mL) [108]. Berry juices obtained directly from frozen fruits of six *V. opulus* genotypes (var. *sargentii*, var. *americanum* and four cultivars, namely “Shukshinskaya”, “Krasnaya Grozd”, “Kiyevskaya Sadovaya” and “P3”) were tested in vitro for DPPH-radical scavenging capacity and antioxidant activities (ABTS, FRAP, ORAC), with the results indicating that *V. opulus* var. *sargentii* exhibits the highest antioxidant potential among all of the investigated genotypes [106].

Oxidative stress is a key driver of inflammation; often, extracts or compounds from IAPS berries exhibit both antioxidant and anti-inflammatory properties. The methanol extract of *B. thunbergii* var. *atropurpurea* roots showed moderate anti-inflammatory activity tested by the COX-1 and COX-2 enzyme (cyclooxygenases) inhibitory assay, while the pure isolates obtained by purification indicated higher activity [98]. The methanol fruit extract of *C. horizontalis* exhibited moderate anti-inflammatory activity, in a dose-dependent way, against lipoxygenase and hyaluronidase, which are key enzymes involved in inflammation; this activity was attributed to the polyphenols (−)-epicatechin, quercetin, and chlorogenic acid [109]. The fruit extract of *E. umbellata* demonstrated anti-inflammatory effects by increasing the production of metalloproteinases, TIMP-1 (Tissue inhibitor of metalloproteinases-1), and MMP-9 (Matrix metalloproteinase-9), and by increasing the expression of brain-derived neurotrophic factor (BDNF), which makes this extract suitable for alleviating neuro-inflammatory conditions [63]. The ethanol extract of the raw (unripe) fruits of *L. ferocissimum* exhibited significant anti-inflammatory activity at a concentration of 31.25 µg/mL in all of the experiments performed; this was determined by measuring the level of inflammation parameters, such as TNF-α, IFN-γ, prostaglandin E2 (PGE2), and nitric oxide (NO) after lipopolysaccharide (LPS) was added to macrophage Raw 264.7 cell line [94]. The aqueous extract obtained from *N. domestica* fruits showed anti-inflammatory activity at concentrations of 1∼10 μg/mL in a dose-dependent way by suppressing the expression of COX-2 and the production of PGE2 in LPS-stimulated human pulmonary epithelial A549 cells without affecting COX–1 expression and COX activity [56]. In vivo studies regarding the anti-inflammatory effects of the ethanol extract obtained from dried fruits of *S. terebinthifolia*, which were conducted using the mouse paw edema method, showed that the extract significantly decreased the mouse paw edema compared to that of the control, but to a lower extent compared to that of the reference (indomethacin) [49]. Another in vivo study regarding the anti-inflammatory potential of the extract obtained from *S. elaeagnifoliumon* dried fruits, using 1% carrageenan-induced paw edema in rats, confirmed that the ethanol extract at 400 mg/kg showed an anti-inflammatory effect (86%) comparable to the effect of indomethacin (92%) [110].

Compounds exhibiting antioxidant properties may show anti-cholinesterase activity, due to some common molecular pathways, with the oxidative stress also accelerating the damage of the cholinergic neurons, so that the antioxidant therapy may be efficient for improving cognitive impairment [63]. Anti-cholinesterase inhibitors are promising agents in the treatment of neurodegenerative diseases. Several studies have been investigating the acetylcholinesterase (AChE) and butyrylcholinesterase (BChE) inhibitory effects of extracts from IAPS berries. Khan et al. [111] showed that two compounds (Horizontoate A and Horizontoate B) isolated from the extract of dried whole plant of *C. horizontalis* exhibited inhibitory potential against both AChE and BChE, with IC_50_ values 1.54–3.41 μM against AChE and 5.97–6.84 μM against BChE. Another study conducted on *C. pannosus* fruits did not find an AChE inhibitory effect, but demonstrated inhibitory effects on other key enzymes of neurotransmitter metabolism, such as monoamine oxidase A (MAO-A) and tyrosinase (TYR), the activities of which were lower than those of the references used in the study (clorgyline and kojic acid, respectively) [112]. The invasive plant *E. umbellata* contains alkaloids, such as galantamine and rivastigmine, which are well-known agents approved to treat Alzheimer’s disease [63]. The fruits of *E. umbellata* contain other bioactive compounds responsible for anti-cholinesterase activity, as demonstrated both in vitro and in vivo; these include phenolic acids (chlorogenic acid, ellagic acid, and gallic acid) and phloroglucinol [63]. The in silico study of Prakash et al. [113] showed that twenty constituents, particularly lantic acid in *L. camara* berries, were good suppressers of human AChE and human carbonic anhydrase II (hCA-II). A neuroprotective effect of the methanol extract of *S. ebulus* fruits was described in the brain of young chickens, with the fruit extract indicating a significant antiemetic effect at 100 mg/kg comparable to that of the metoclopramide [105].

In the search for plant products with anticancer properties, IAPS berries, particularly the unripe fruits, can be a promising resource. Thus, extracts or compounds obtained from several IAPS fruits showed cytotoxic properties, most of which were evaluated by the in vitro MTT assay, as follows: (1) the ethanol fruit extract of *C. orbiculatus* exhibited significant cytotoxicity against human melanoma A375-S2 and human cervical carcinoma Hela cells, as authors isolated four sesquiterpenoids, which are responsible for this pharmacological effect [114]; (2) the ethanol extract from unripe fruits of *H. helix* suppressed the cell migration in rat prostate cancer cell lines (Mat-LyLu cells, strongly metastatic) and inhibited proliferation of rat prostate cancer cell lines (AT-2 cells, weakly metastatic) [51]; the ethanol extract from unripe fruits of *L. ferocissimum* exhibited strong cytotoxic activity on the Du145 cell line (ATCC HTB-81TM, human prostate cancer) and the A549 (ATCC CCL-185, human lung cancer) cell lines [94]; the authors showed that the cytotoxic activity was stronger than that of the extracts from ripe fruits or leaves tested on the same cell lines; (3) the alkaloid nandsterine isolated from the fruits of *N. domestica* showed cytotoxicity against human leukemia HL–60 cells [56]; (4) the ethyl acetate fraction of extracts obtained from dried fruits of *R. typhina* demonstrated an antiproliferative effect on human colon cancer HT29 cells, with the compounds luteolin and luteolin-7-O-glucuronide being identified as the main antiproliferative agents [115]; (5) the essential oil obtained from *S. terebinthifolius* berries exhibited antitumor properties against human breast cancer MCF-7 cell lines [116]; (6) the polyphenol-rich extract from *S. nigrum* fruits showed strong cytotoxicity and induced apoptosis in prostate cancer cells CA-HPV-10 [117]; (7) the methanol extract obtained from fresh fruits of *V. opulus* decreased the cell viability of colon cancer cell line Lovo up to 52.06% in a concentration-dependent way [118].

Regarding the antimicrobial activity of extracts/compounds from IAPS berries, there is a great variability even within the same species, due to different preparations and composition of extracts, with the last varying in a way that is strongly related to the environmental factors (origin, climate, harvest season, maturity, etc.). Most studies reported the results in terms of bacterial growth inhibition zones, but also in terms of minimum inhibitory concentrations (MICs) using pathogenic microbial standard strains (ATCC collection). The majority of the results showed weak or moderate antimicrobial activities of fruit extracts, particularly when compared to those of classic antibiotics, with the extracts generally being more active against Gram-positive bacteria (*Staphylococcus aureus*, *Streptococcus* spp.), which supports their traditional use in wound healing. Examples of antimicrobial extracts are as follows: various extracts of *B. thunbergii* berries showing moderate activity [119]; methanol extract of *L. camara* fruits that produced inhibition zones ranging between 9 and 12.3 mm against *S. aureus, Micrococcus luteus, Salmonella setubal, E. aerogenes, K. pneumoniae,* and *E. coli* [120]; the ethanol extract of *P. americana* fruits, which showed MIC values ranging from 25 to 200 µL/mL, with the activity being mostly attributed to the content of betalains, catechins, and gallic acid [103]; the ethanol extract of *R. typhina* fruits that produced a higher inhibitory effect on *Bacillus cereus* and *Bacillus subtilis* (inhibition zones of 11–20 mm depending on the extract concentration, MIC values of 0.10 and 0.20%) [104]; the concentrated extract obtained from *R. typhina* dried fruits, which indicated strong activity against *Streptococcus pyogenes* ATCC 19615 (inhibition zone of 20 mm) [107]; the ethanol extract of dried fruits of *S. ebulus* that inhibited *B. subtilis*, *Enteroccocus fecalis*, *S. aureus*, and *Pseudomonas fluorescens*, particularly when the well diffusion test was performed compared to the disk diffusion test [121]; the ethanol extract from dried fruits of *S. terebinthifolia*, which had an inhibitory effect on *E. coli*, *S. aureus* and *C. albicans* (inhibition zones of 17, 14 and 12 mm, respectively) [49]; the solanine alkaloid extracted from the unripe fruits of *S. dulcamara*, which inhibited the growth of *E. coli* and *S. aureus* [122]; the methanol extract of unripe fresh fruits of *S. viarum* at 100 μg concentration, which had a good inhibitory effect on *P. aeruginosa* and *B. subtilis* (inhibition zones of 21 and 12 mm, respectively, according to the well diffusion test) [123]; and the fruit extract of *V. opulus*, which demonstrated remarkable activity against *Cronobacter muytjensii* and *P. aeruginosa* (inhibition zones of 28.6 and 22.4 mm, according to the well diffusion test, and MIC values of 0.24 μL/mL) [124]. However, the antimicrobial activity of fruit extracts has been less studied compared to that of other parts of the plant, such as the roots, leaves, and essential oils extracted from the fruit.

Some of the investigated IAPS extracts showed anti-diabetic properties in either in vitro or in vivo studies, as follows: (1) the in vitro study conducted through the α-amylase inhibition test on *C. pannosus* fruits showed IC_50_ values of the polar and apolar fruit extracts of 27.82 and 12.74 µg/mL, respectively, which were lower than those for acarbose (381.08 µg/mL), a reference inhibitor used to treat and prevent type 2 diabetes [112]; (2) extracts obtained from the berries of *E. umbellata* showed strong inhibitory effects on enzymes involved in glucose metabolism (α-amylase, α-glucosidase) and an increase in the expression of the hormone adiponectin [63]; (3) the mucilage extracted from dried aerial parts of *C. horizontalis* orally administered to rats at dose of 250 mg/kg b.wt. exhibited both hypoglycemic and antidyslipidemic activities, demonstrated by an improvement in glucose tolerance, body weight gain, blood glucose, glycated hemoglobin, liver glycogen, serum lipid profile, and atherogenic risk factors [125]; (4) freeze-dried fruits of *L. obtusifolium* administered as a powder at concentration of 1–2% in the diet of streptozotocin (STZ)-induced diabetic rats fed a high-fat diet for 6 weeks may regulate STZ-induced pancreatic and renal damages as consequences of hyperglycemia and hyperlipidemia through the increase in insulin secretion [126]; very significant anti-diabetic properties were demonstrated in both in vitro and in vivo studies (Oral Glucose Tolerance Test in rats, pancreatic α-amylase, pancreatic α-glucosidase) by the ethanol extracts obtained from the fruit powder of *S. elaeagnifolium* [110].

Pietrzyk’s study, based on the investigation of *V. opulus* berry fresh juice and a polyphenol-rich fraction in relation to their biological activities after the in vitro mouth–gastric–intestine digestion, indicates that both digested samples exhibit bioactivities by using different cellular models (myoblasts L6 cell line, hepatocytes HepG2 cell line, insulinoma β cells MIN6 cell line, and adipocytes 3T3-L1 cell line). Their results showed that both samples stimulated glucose uptake, decreased lipid accumulation (in L6 myoblasts, HepG2 hepatocytes), and decreased the secretion of inflammatory cytokines (in 3T3-L1 adipocytes) [127]. However, the authors concluded that further investigations are required before the *V. opulus* berries can be used as a dietary supplement because for all digested and non-digested samples, they noted a lipotoxic effect against pancreatic β MIN6.

Although pokeweed (*P. americana*) berries are considered toxic [38,43,128,129,130], studies on their food use continue to be published, mostly based on their confirmed bioactive properties. One example includes the application of a betalain extract obtained from dried *P. americana* fruits in a cheese formulation as a phytochemical-enriched and antioxidant food product [131]. Pokeweed berry extracts were proven to possess the following pharmacological properties: strong in vitro antioxidant properties [103,132]; anxiolytic effects for both fresh and dried berries, as confirmed in a zebrafish model by alleviating scopolamine-induced anxiety; inhibition of acetylcholinesterase activity; an increase in antioxidant enzymes’ activity and a decrease in lipid and protein peroxidation [133]; antimicrobial activity against *Staphylococcus aureus* ATCC 6538/MRSA 1263, *Bacillus subtilis* 12488, *Klebsiella pneumoniae* 11 [103], and *Escherichia coli* [130]; strong antiproliferative and apoptotic activities against HCT-116 colon cancer cells [134]. Marinas et al. [103] conducted a comparative study of the biological activities of leaves and berry extracts of *P. americana*, showing that the berry extract was more active against bacterial strains than the leaf extract. They concluded that this was probably due to the presence of betalains, catechins, and gallic acid—compounds which were not detected in the leaf extract through the HPLC analysis. The example previously mentioned emphasizes the need to study the pharmacological properties not only with regard to the therapeutic effects of berry extracts, but also their potential toxicity and side effects.

Toxicity issues within, IAPS berries have been documented in the published literature, and the results are reported in Table 3. The information regarding the safety of plant species reported in the international INCHEM database—an International Program on Chemical Safety (IPCS) of the World Health Organization (WHO) is described in Table 3, e.g., for *H. helix*, *I. aquifolium*, and *S. nigrum*. Photos of fruits have also been included in Table 3; these were sourced from the Invasive Plant Atlas, GBIF, or CABI compendium.

Numerous IAPS produce fruits that might be toxic to humans or animals if ingested, particularly when unripe. According to the information provided in Table 3, about 68% of the described invasive plants produce potential toxic berries. Most of them are believed to be toxic based on poisoning case reports and not on in vitro/in vivo toxicity studies, with some of them producing mild symptoms, while others are very toxic (*Solanum* spp.). The invasive plants identified as having edible fruits are *A. asparagoides*, *B. thunbergii*, *E. umbellate*, and *R. typhina*, while others (*R. armeniacus*, *L. obtusifolium*, *C. sericea*, *C. orbiculatus*, *A. glandulosa* var. *brevipedunculata*) could not be classified according to toxicity due to the lack of published research literature. I found few scientific papers that tackled the subject of establishing safe doses of fruits from IAPS, with most of them reporting general toxic effects and symptoms without scientific confirmation. Here, I provide some examples of the reported quantitative toxicity data: (i) methanol extract obtained from fresh berries of the autumn olive (*E. umbellata*) was found to be safe in quantities of up to 10 mg/kg in mice [135]; (ii) the protein ebulin f, isolated from green fruits of the dwarf elderberry (*S. ebulus*), was considered to be toxic at doses of 1.4 mg/kg i.p. in mice [136]; (iii) the ethanol extract of silverleaf nightshade (*S. elaeagnifolium*) fruits was determined to be toxic and caused the death of mice at a dose of 2000 mg/kg (oral administration) [137]; (iv) a lethal dose (LD_50_) of the fruit juice of guelder rose/European cranberrybush (*V. opulus*) was found to be over 2000 mg/kg in acute tests on rats and mice [138]. For other (and most) IAPS berries, scientific confirmation of their acute and long-term toxicity is awaited as a basis for their potential uses in foods or pharmaceuticals.

The toxic compounds found in fruits of IAPS display various chemical structures, mainly belonging to the following classes of compounds: steroidal alkaloids (e.g., solanine, solasodine, solamargine, chaconine) that are frequently distributed within the Solanaceae family; tropane alkaloids; steroidal glycosides (e.g., steroidal/triterpene saponins, phytolaccoside A, B, D, E, and G); cyanogenic glycosides; lectins; triterpenoids (lantadene); or tannins (which have an antinutritive effect) [128,137,139,140]. Despite its nutritional value for ruminant livestock [140] and its several confirmed pharmacological properties, e.g., anticholinesterase and antimicrobial properties [113,120], the bunch berry (*L. camara*) is listed among the 10 most widely distributed invasive alien species [25] and is among the most toxic invasive plant species. The toxins of *L. camara* occur in its leaves and green unripe fruits, and are classed as pentacyclic triterpenes and triterpenoids (lantadenes)—toxic compounds that mainly cause photosensitization and liver damage [140].

**Table 3:** Summarized information on berries of IAPS: fruit characteristics, pharmacological activity, and toxicity.

Scientific Name	Fruit Characteristics	Ref.	Pharmacological Activity and Toxicity of Fruits		Ref.
*Ampelopsis glandulosa var. brevipedunculata*	Small berries (6–8 mm in diameter) in small clusters, yellow, green, purple or blue with small white and gray spots (black when ripe) 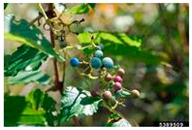 Photo source: https://www.invasive.org/browse/detail.cfm?imgnum=5389509 (accessed on 12 February 2025)	[36]	Pharmacological activity	Antioxidant, antiphlogistic, depurative and febrifuge, anti-hepatotoxic, and weak antibacterial activity. Used externally for the treatment of boils, *Herpes zoster*, abscesses, ulcers, traumatic bruises, aches, and insect stings.	[43,96,141,142]
			Toxicity to humans	No identified reports of fruit toxicity.	
*Asparagus asparagoides*	Globular berries (6–10 mm wide) with 0–4 seeds, green (red when ripe) 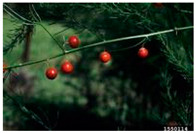 Photo source: https://www.invasive.org/browse/detail.cfm?imgnum=1550114 (accessed on 12 February 2025)	[53]	Pharmacological activity	Mentioned only as a remedy for stomach disorders (South African traditional medicine); no other data available for fruits.	[53]
			Toxicity to humans	No identified report on fruit toxicity; according to [143], the fruits are unpalatable, but not reported as toxic.	[143]
*Berberis thunbergii*	Small egg-shaped berries (7–10 mm long), single or in clusters, bright red 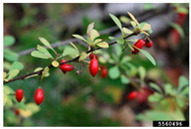 Photo source: https://www.invasive.org/browse/detail.cfm?imgnum=5560496 (accessed on 12 February 2025)	[36]	Pharmacological activity	Antioxidant, antibacterial, anti-inflammatory, antihypertensive, and hepatoprotective.	[98,119,144]
			Toxicity to humans	Barberry fruits are considered edible.	[42,144]
*Celastrus orbiculatus*	Rounded berries, 1 to 3 clustered in the leaf axils, green during summer, yellow to orange in fall; 3-section capsules with orange-red seeds. 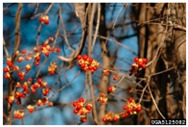 Photo source: https://www.invasive.org/browse/detail.cfm?imgnum=5125082 (accessed on 12 February 2025)	[36]	Pharmacological activity	Antioxidant, inhibitor of nitric oxide production, anti-melanoma activity, anti-thrombus effect, and anti-rheumatoid arthritis.	[58,114,145,146,147]
			Toxicity to humans	No identified report on fruit toxicity.	
*Cestrum nocturnum*	Small berries (8–10 mm in diameter); white or aubergine; hard or juicy. 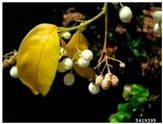 Photo source: https://www.invasive.org/browse/detail.cfm?imgnum=5419395 (accessed on 12 February 2025)	[92]	Pharmacological activity	No available data on fruits; only on leaves and flowers.	
			Toxicity to humans	Known toxicity to humans, particularly in the case of unripe berries, due to diterpene- and steroid-glycosides.	[92]
*Cornus sericea* *(Cornus stolonifera)*	Berries in flat clusters (4–10 mm in diameter); white with a green tinge. 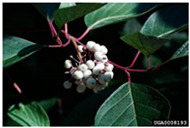 Photo source: https://www.invasive.org/browse/detail.cfm?imgnum=0008193 (accessed on 12 February 2025)	[36]	Pharmacological activity	Antioxidant.	[99]
			Toxicity to humans	No identified reports of fruit toxicity.	
*Cotoneaster franchetii*	Elliptic berries (6–7 mm in diameter); orange to red or black; containing 3–5 seeds. 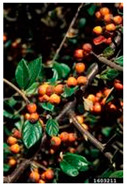 Photo source: https://www.invasive.org/browse/detail.cfm?imgnum=1603211 (accessed on 12 February 2025)	[36]	Pharmacological activity	Antioxidant activity of other Cotoneasters spp. fruits.	[70]
			Toxicity to humans	Most cotoneasters fruits are mildly toxic, due to cyanogenic glycosides (prunasin, amygdalin). Studies on the fruits of some Cotoneasters spp. (*Cotoneaster divaricatus*) indicated a safe dose of 0.5 g/kg in rats; the first symptoms of cyanide toxicity occur at a dose of 1 g/kg.	[70,71]
*Cotoneaster horizontalis*	Small sub-globose or ellipsoid berries (5–7 mm in diameter); bright red; berries contain 3 seeds. 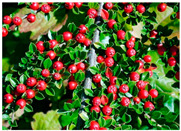 Photo source: https://www.cabidigitallibrary.org/doi/10.1079/cabicompendium.16870 (accessed on 12 February 2025)	[69]	Pharmacological activity	Antioxidant, anti-inflammatory, anti-diabetic, and anti-dyslipidemic; cholinesterase inhibitor.	[109,111,125,148]
			Toxicity to humans	Most cotoneasters fruits are mildly toxic due to cyanogenic glycosides (prunasin, amygdalin). Studies on the fruits of some Cotoneasters spp. (*Cotoneaster divaricatus*) indicated a safe dose of 0.5 g/kg in rats; the first symptoms of cyanide toxicity occur at a dose of 1 g/kg.	[70,71]
*Cotoneaster lacteus*	Small red berries in clusters. 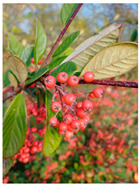 Photo source: https://observation.org/photos/80076609.jpg (accessed on 12 February 2025)	[71]	Pharmacological activity	Antioxidant.	[71]
			Toxicity to humans	Most cotoneasters fruits are mildly toxic due to cyanogenic glycosides (prunasin, amygdalin). Studies on the fruits of some Cotoneasters spp. (*Cotoneaster divaricatus*) indicated a safe dose of 0.5 g/kg in rats; the first symptoms of cyanide toxicity occur at a dose of 1 g/kg.	[70,71]
*Cotoneaster pannosus*	Globose or ovoid berries (7–8 mm in diameter); dark red with 2 seeds. 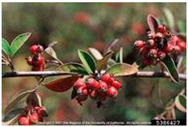 Photo source: https://www.invasive.org/browse/detail.cfm?imgnum=5386427 (accessed on 12 February 2025)	[112]	Pharmacological activity	Antioxidant, anti-diabetic, and neuroprotective.	[112]
			Toxicity to humans	Most cotoneasters fruits are mildly toxic due to cyanogenic glycosides (prunasin, amygdalin). Studies on the fruits of some Cotoneasters spp. (*Cotoneaster divaricatus*) indicated a safe dose of 0.5 g/kg in rats; the first symptoms of cyanide toxicity occur at a dose of 1 g/kg.	[70,71]
*Elaeagnus umbellata*	Small, round berries; orange to red with silvery or coppery spots, with seeds. 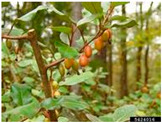 Photo source: https://www.invasive.org/browse/detail.cfm?imgnum=5424016 (accessed on 12 February 2025)	[36]	Pharmacological activity	Antioxidant, inhibits fat accumulation, anti-inflammatory, anticancer, supports immune system, antiviral, antifungal, gastroprotective, anti-diabetic, anti-cholinesterase activity, anti-amnesic, and anti-diarrheal.	[63,64,100,149]
			Toxicity to humans	Fruits are considered edible; fruit extract was found to be safe in quantities up to 10 mg/kg (mice).	[135]
*Hedera helix*	Globose berries; dark blue to black; fleshy outer layer and stone-like seeds. 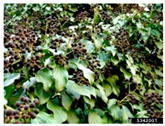 Photo source: https://www.invasive.org/browse/detail.cfm?imgnum=5342007 (accessed on 12 February 2025)	[36]	Pharmacological activity	Anticancer and anti-helminthic.	[51]
			Toxicity to humans	Acute toxicity resulting in intoxication; fruits are toxic in high doses (gastric disturbances), producing eczema through skin contact due to hedera-saponins that decompose to toxic hederin compounds. Local digestive irritant damage (IPCS INCHEM database).	[38,43,150]
*Ilex aquifolium*	Spherical berries (7–8 mm in diameter) in small clusters; bright red to orange and yellow; each berry contains 4 seeds. 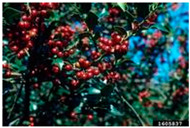 Photo source: https://www.invasive.org/browse/detail.cfm?imgnum=1605837 (accessed on 12 February 2025)	[38]	Pharmacological activity	Antioxidant (seeds of the fruits); no medicinal use.	[151]
			Toxicity to humans	Fruits are toxic to children and young people due to ilicin and cyanogenic glucosides. Gastrointestinal symptoms; 20–30 berries are considered to be a “lethal dose” (IPCS INCHEM database).	[38,43,152]
*Lantana camara*	Spherical (3 mm in diameter); shiny blue; black drupes; green berries when unripe. 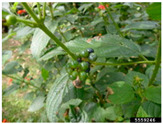 Photo source: https://www.invasive.org/browse/detail.cfm?imgnum=5559249 (accessed on 12 February 2025)	[43]	Pharmacological activity	Inhibitors of human acetylcholinesterase, carbonic anhydrase II, and carboxylesterase; antibacterial properties.	[113,120]
			Toxicity to humans	Green berries were reported to be particularly toxic (hepatotoxic, nephrotoxic). Some other studies showed that toxicity was not significant in children.	[42,43,153]
*Ligustrum obtusifolium*	Sub-globose to broadly ellipsoid small berries (5–8 × 6 mm) in terminal clusters; purple-black. 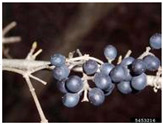 Photo source: https://www.invasive.org/search/action.cfm?q=Ligustrum%20obtusifolium (accessed on 12 February 2025)	[65]	Pharmacological activity	Antihyperglycemic.	[126]
			Toxicity to humans	No literature data on toxicological risk. Berries may induce digestive problems due to terpenoid glycosides (ligustrin).	[154]
*Lycium ferocissimum*	Globular or ovoid berries (10 mm in diameter); green or orange to red (ripe). 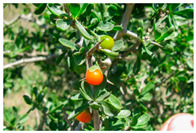 Photo source: https://www.cabidigitallibrary.org/doi/10.1079/cabicompendium.31903 (accessed on 12 February 2025)	[93]	Pharmacological activity	Antioxidant, anti-inflammatory, cytotoxic activity on the Du145 (human prostate cancer) and A549 (human lung cancer) cell lines.	[94]
			Toxicity to humans	Berries are poisonous if ingested.	[155]
*Nandina domestica*	Round berries (5–10 mm in diameter) in clusters; bright red; containing 2 seeds. 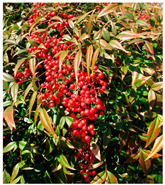 Photo source: https://www.cabidigitallibrary.org/doi/10.1079/cabicompendium.35692 (accessed on 12 February 2025)	[156]	Pharmacological activity	Antioxidant, antitumor, and anti-inflammatory effects; protective effects on liver and kidney toxicity induced by arsenic trioxide.	[56,101,157,158]
			Toxicity to humans	Low toxicity. Mild clinical effects (vomiting, abdominal pain, diarrhea, and nausea) occur if raw berries are ingested due to the presence of cyanogenic glycosides.	[38,56,156]
*Parthenocissus quinquefolia*	Berries (1–1.2 cm in diameter) on long-stemmed clusters; bluish black. 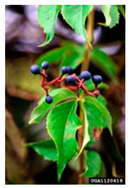 Photo source: https://www.invasive.org/browse/detail.cfm?imgnum=1120419 (accessed on 12 February 2025)	[36]	Pharmacological activity	Antioxidant.	[102,159]
			Toxicity to humans	Berries are considered toxic due to oxalic acid, which is also present in high concentrations in leaves.	[43,97,159]
*Phytolacca americana*	Round berries (0.64 cm wide); deep purple to almost black; each containing 10 seeds (2.5–3.0 mm in diameter). 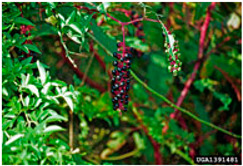 Photo source: https://www.invasive.org/browse/detail.cfm?imgnum=1391481 (accessed on 12 February 2025)	[160]	Pharmacological activity	Antioxidant, antimicrobial, inhibitor of tyrosinase and xanthin oxidase; green fruits have purgative effects; when separated from the fruits, the seeds exhibited anticancer properties against human colon cancer, which was mainly attributed to the benzodioxine “americanin A”. Anxiolytic effects in a zebrafish (*Danio rerio*) model (alleviated Scopolamine-induced anxiety; enhanced cognitive performance; inhibited AChE activity; supported antioxidant defense mechanisms).	[103,132,133,134,161]
			Toxicity to humans	Berries are toxic if ingested (causing abdominal pain, nausea, vomiting, and diarrhea).	[38,43,128,129,130]
*Prunus serotina*	Berries (8–10 mm in diameter); dark red to black. 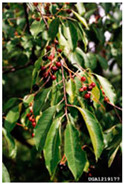 Photo source: https://www.invasive.org/browse/detail.cfm?imgnum=1219177 (accessed on 12 February 2025)	[43]	Pharmacological activity	Antioxidant and antihypertensive.	[74]
			Toxicity to humans	Berries are toxic due to cyanogenic glycosides.	[43]
*Rhus typhina*	Round, hairy, dark red fruits (berrylike drupes) clustered in 6–10′’ spikes. 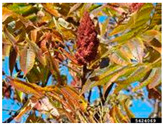 Photo source: https://www.invasive.org/browse/detail.cfm?imgnum=5424069 (accessed on 12 February 2025)	[36,46]	Pharmacological activity	Antioxidant, antimicrobial, anti-inflammatory, anti-hemorrhoidal, antiseptic, diuretic, anticancer, hepatoprotective, and anti-streptococcal activity, and weak antiproliferative potential with regard to the HepG2 cell line.	[104,107,115,162,163]
			Toxicity to humans	Berries are considered edible, as they are used in food products (meat, cheese, and drinks). Antinutritive effects might occur due to high levels of tannins. Low hemolytic activity on sheep red blood cells; fruit extract was found to be biocompatible with human gingival fibroblasts (HGFs).	[47,107]
*Rubus armeniacus* *(Rubus bifrons)*	Round black, shiny drupelets (2 cm aggregates); each drup contains one seed. 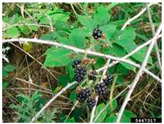 Photo source: https://www.invasive.org/browse/detail.cfm?imgnum=5447317 (accessed on 12 February 2025)	[14]	Pharmacological activity	Antioxidant.	[164,165]
			Toxicity to humans	No identified report of fruit toxicity.	
*Sambucus ebulus*	Small glossy berries; black. 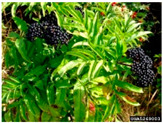 Photo source: https://www.invasive.org/browse/detail.cfm?imgnum=5269003 (accessed on 12 February 2025)	[43]	Pharmacological activity	Antioxidant, antidepressant, antiarthritic, anti-inflammatory, antimicrobial, cytoprotective, antiemetic, and neuroprotective effects.	[105,121,166]
			Toxicity to humans	Green/unripe fruits are toxic due to the presence of a lectin (ebulin); fruit extracts showed no toxicity up to 2 g/kg bw (intraperitoneally, in mice), with the exception of the ethyl acetate extract, which showed severe toxicity.	[108,167]
*Schinus terebinthifolia*	Small drupes in dense clusters; 4–5 mm in diameter; bright red, with seeds. 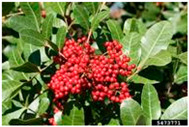 Photo source: https://www.invasive.org/browse/detail.cfm?imgnum=5473771 (accessed on 12 February 2025)	[48]	Pharmacological activity	Antioxidant, antimicrobial, and antitumor effect of berry essential oil; anti-inflammatory effect.	[49,116]
			Toxicity to humans	Toxic and allergic reactions occur as a result of ingestion (due to moronic acid).	[168]
*Solanum carolinense*	Globular, pulpy, juicy, and smooth berries (8–20 mm in diameter), with 40–170 seeds; immature berries are green, while the mature ones are yellow to orange. 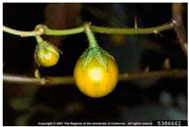 Photo source: https://www.invasive.org/browse/detail.cfm?imgnum=5386662 (accessed on 12 February 2025)	[76]	Pharmacological activity	Antibacterial.	[169]
			Toxicity to humans	Berries are poisonous due to glycoalkaloids; moderate toxicity.	[43]
*Solanum dulcamara*	Ovoid berries in stemmed clusters; green; red at maturity. 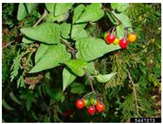 Photo source: https://www.invasive.org/browse/detail.cfm?imgnum=5447273 (accessed on 12 February 2025)	[170]	Pharmacological activity	Antioxidant, antimicrobial, and anticancer properties.	[117,122]
			Toxicity to humans	Immature berries are particularly poisonous due to steroidal alkaloids (causing gastrointestinal disorders and neurologic, cardiovascular, and respiratory symptoms).	[43,171]
*Solanum elaeagnifolium*	Globose berries (0.8–1.4 cm in diameter); marbled green, yellow and orangish brown (ripe). 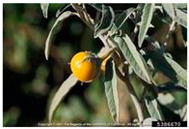 Photo source: https://www.invasive.org/browse/detail.cfm?imgnum=5386670 (accessed on 12 February 2025)	[81]	Pharmacological activity	Antioxidant, anti-diabetic, anti-inflammatory, antifungal, and anticancer properties. Solasodine extracted from fruits is used for the production of corticosteroid hormones.	[81,108,110,172]
			Toxicity to humans	Fruit extract is toxic (2000 mg/kg caused death in mice).	[137]
*Solanum mauritianum*	Globose berries (10–15 mm in diameter) in compact terminal clusters; green; yellow (ripe). 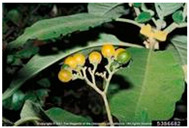 Photo source: https://www.invasive.org/browse/detail.cfm?imgnum=5386682 (accessed on 12 February 2025)	[83]	Pharmacological activity	Solasodine extracted from fruits is used as an anti-inflammatory and for the production of corticosteroid hormones.	[67,173]
			Toxicity to humans	Green berries are very toxic.	[43,83]
*Solanum nigrum*	Globular berries (5–13 mm in diameter) with woody seeds; dark green; black (ripe). 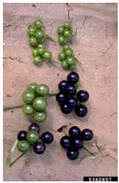 Photo source: https://www.invasive.org/browse/detail.cfm?imgnum=5362857 (accessed on 12 February 2025)	[86]	Pharmacological activity	Antioxidant and antimicrobial properties; protection against induced kidney damage; hepatoprotective, anti-diabetic, anti-ulcer, cardioprotective, analgesic, and anti-inflammatory effects; anticancer properties against HeLa cell line.	[67,174,175,176]
			Toxicity to humans	Unripe berries are toxic due to the presence of solanine and other glycol-alkaloids (chaconine and solasodine), causing digestive, neurologic, respiratory and cardiac symptoms; ripe berries are less toxic. Immature fruit extracts are strongly dose-dependently cytotoxic and induce significant DNA damage in human lymphocytes (comet assay). Included in the IPCS INCHEM database.	[38,43,86,177,178,179]
*Solanum pseudocapsicum*	Globose berries (1–2 cm wide); red to yellow, with seeds. 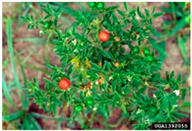 Photo source: https://www.invasive.org/browse/detail.cfm?imgnum=1392055 (accessed on 12 February 2025)	[87]	Pharmacological activity	Antioxidant; anti-tumor.	[180]
			Toxicity to humans	Berries are poisonous due to solanocapsine, causing central anticholinergic syndrome. Cytotoxic.	[87,180]
*Solanum seaforthianum*	Globose berries (0.8–1.4 cm in diameter); bright, shiny red when ripe, with seeds (4–20 per berry). 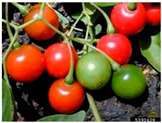 Photo source: https://www.invasive.org/browse/detail.cfm?imgnum=5390428 (accessed on 12 February 2025)	[88]	Pharmacological activity	Antihelminthic.	[181,182]
			Toxicity to humans	Berries are very toxic due to the presence of glycoalkaloids.	[183]
*Solanum viarum*	Globose berries (2–3 cm across) with ~400 seeds; green to yellow (ripe). 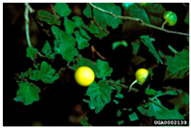 Photo source: https://www.invasive.org/browse/detail.cfm?imgnum=0002139 (accessed on 12 February 2025)	[91]	Pharmacological activity	Antioxidant, antipyretic, antibacterial, insecticidal, analgesic, anticancer, and antimicrobial activity.	[123,184,185]
			Toxicity to humans	Unripe berries are toxic due to the presence of solasodine.	[185]
*Viburnum opulus*	Rounded berries in bunches, with 1 flattened seed; red. 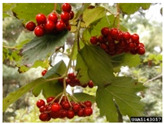 Photo source: https://www.invasive.org/browse/detail.cfm?imgnum=5143057 (accessed on 12 February 2025)	[36,127]	Pharmacological activity	Antioxidant, antimicrobial, anti-diabetic, anti-obesity, and anticancer properties; prevention of hepatic lipotoxicity; adipogenesis regulation.	[106,118,124,127,186]
			Toxicity to humans	Berries cause mild symptoms in humans when ingested. Berry juice: the lethal dose (LD_50_) was over 2000 mg/kg (rats, mice; acute test).	[36,138]

As described in Table 3, the fruits of selected berry-producing IAPS may offer great potential for pharmaceutical/medicinal applications due to their specific bioactivities. Since the mechanical removal of IAPS remains the most common eradication measure, the use of the biomass left after removal or collection of IAPS fruits may suppress plant expansion. However, further evaluation of their toxicity via in vitro and in vivo studies is required. Usually, such plants produce toxins as a survival mechanism and in response to different factors (defense against other plants—allelopathy; defense against herbivores; or for self-defense or competition). Studies have confirmed strong biogeographical differences regarding toxins between a plant’s native area and the newly invaded areas, sometimes indicating potentially greater toxicity of the plant in the invaded region, e.g., for *Senecio pterophorus* [187], so evaluation of toxicity should be performed for every individual case. This will provide a lot of opportunity for research in this direction.

The findings gathered in Table 3 can help us gain insights into challenges and opportunities to accelerate research or innovation in this field. Although IAPS fruits continue to be used traditionally and in home-made products, few commercial IAPS berry products were identified on the global market (e.g., sumac juice from berries of *R. typhina* staghorn sumac/Magasin Ferme et Forêt Store, or *Phytolacca* Berry Q Herbal Mother Tincture). However, different purified compounds isolated from IAPS have already found commercial applications, e.g., a mixture of purified solasodine glycoalkaloids from *S. elaeagnifolium*, which is applied in the formulation of the cream Curaderm BEC5 used against non-malignant and malignant skin lesions [2]. A published patent describes an application of the *R. typhina* fruit extract as anti-aging drug or healthcare food [188]. An interventional clinical study (NCT04069286) sponsored by the Infan Industria Quimica Farmaceutica Nacional has begun, focusing on the “Efficacy of oral aroeira (*Schinus terebinthifolia raddi*) compared to omeprazole in dyspepsia: a randomized and double-blind study”. Another phase 2 clinical study (NCT02467543) sponsored by the University of Johannesburg is underway, titled, “The Efficacy of *Viburnum opulus* 3X in the Treatment of Primary Dysmenorrhea”.

Occurrence of a chemical compound of concern for human health in a given part of the plant does not necessarily indicate that the final herbal product is as toxic, because the toxicity ultimately depends on the preparation mode of the final product. Botanical products to be used in foods or pharmaceuticals must be proven to be safe and effective in well-controlled clinical trials, similarly to individual chemicals (drugs).

As shown in Table 3, the toxic effects of some of the berries of the selected invasive plants occur mainly when the berries are in their unripe form, with several studies demonstrating that the toxic effects decrease with the plant growth, as is the case with *S. nigrum* berries [189]. There are studies reporting that drying and boiling might reduce the toxic effect of *Solanum aculeastrum* berries in male Wistar rats compared to that of the fresh fruits [190], with heat treatment being a potential solution to the safety issues posed by such fruits. Given that most of the toxic compounds of non-edible berries are saponins and alkaloids, several published studies have shown that cooking, soaking, canning, and fermentation may reduce the toxic effects of saponins [191], and high-temperature treatments (steaming, boiling, stir-frying) may reduce the toxic effects of alkaloids, while the use of adjuvants may decrease the toxicity of the heat-resistant toxic alkaloids [192]. Because managing IAPS by eradicating them is enormously costly, the identification of other strategies for utilizing their biomass for the biotechnological and biomedical sector should be performed in a cost–benefit manner, considering both the harmful and positive impacts.

## 6. Conclusions and Future Prospects

Invasive alien plants have typically been associated with negative impacts on ecosystems or health, while fewer studies have focused on potential useful applications.

This review highlighted the historical medicinal uses and critically assessed the new findings on pharmacological properties and available toxicity data of fruits of 35 berry-producing species from 16 families of invasive plants. Some of the berries showed remarkable biological activities, ranging from antioxidant, antimicrobial, anti-inflammatory, and anti-diabetic to anticancer, anti-anxiety, and neuroprotective properties. With regard to toxicity, most studies have focused on various symptoms and disorders caused by fruit ingestion or skin contact; instead, toxicity from in vivo studies has been poorly reported, probably because IAPS toxicity was taken as such based on historical poisoning experiences. Very few commercial IAPS berry products have been identified on the global market, but different isolated compounds have found commercial applications. On the other hand, interventional clinical studies have recently started to demonstrate the efficacy of some IAPS (*Schinus terebinthifolia raddi*, *Viburnum opulus*) in different diseases.

Hopefully, this work will serve as a starting point for further exploration of these plants, with the aim of recovering or discovering valuable phytochemicals responsible for their interesting biological activities, which may have medicinal, cosmetic, or food-related applications. Several steps for further research are proposed, as follows: (1) identification of a particular berry-producing IAPS, in conjunction with rapid detection by deep learning-based systems; (2) collecting information on their regionally based environmental impact score; (3) searching for control measures and eco-friendly eradication methods, if available; (4) obtaining information on the potential medicinal benefits of the identified IAPS; (5) specific/selective extraction of target compounds belonging to different classes, and analysis of their content; (6) in vitro testing and screening for biological activities; (7) identification of the compound(s) responsible for specific bioactivity and elucidation of the mechanism of action, if not reported; and (8) in vivo testing of the developed extract, product or compound, for assessing safety and toxicity.

Cooperating with invasive plants by exploiting different parts of the plant to obtain bioactive extracts and products could be another way to control their spread and could provide numerous opportunities for various industries. Of course, the efforts made by conservation practitioners and policymakers to eradicate them continue to be highly necessary in certain areas, but a context-dependent consideration of these plants’ positive or negative attributes should also be considered.

## Figures and Tables

**Table 1:** Classification, terminology, and explanations for human-accompanying plants used in studies on invasive plant species.

Criteria	Classification into Groups and Subgroups			
General				
Occurrence	Native; Apophytes; (indigenous, autochthonous) native species occurring in native man-made habitats independent of human activities			
	Non-native; Anthropophytes; (alien, non-indigenous, exotic, introduced, adventive, allochthonous, and synanthropic) occur as result of human activities; not always harmful (e.g., crop species)			
Alien				
Residence time (time of appearance)	Archaeophytes Introduced before 1500 A.D., both deliberately or accidentally			
	Neophytes Introduced after 1500 A.D., both deliberately or accidentally			
Means of introduction	Direct; Hemerophytes; (deliberate, intentional)			
	Indirect; Xenophytes; (accidental, unintentional)			
Type of habitat encountered	Ergasiophytes Kept only in cultivation			
	Ergasiophygophytes Kept in cultivation, but with occasional escape			
	Ergasiolipophytes Formerly planted, currently occurring in the territory in question without the need for human intervention			
	Ephemerophytes Occur temporarily in man-made habitats			
	Epekophytes Established in man-made habitats, some of them could become invasive			
	Neoindigenophytes Established in the region, occur in man-made habitats, penetrate to natural habitats			
Invasion status (plants outside cultivation)	Casual; (sub-spontaneous, occasional, ephemeral) May grow outside the cultivation area but do not form self-sustaining populations in the invaded area, relying on repeated introductions			
	Naturalized; (established) form self-sustaining and reproducing populations in the new area without the need for human intervention		Invasive; Agryophytes; weeds spread rapidly over a large area, threatening biodiversity and impacting the economy and humans in general	
			Non-invasive; weeds do not currently reproduce and spread as invasive plants in a given area	
Impact	Ecological	EICAT * scoring of the environmental impact		Minimal concern (MC)
	Socio-economic			Minor (MN)
	Health			Moderate (MO)
				Major (MR)
				Massive (MV)
Purpose of plant introduction	Ornamental			
	Food			
	Medicinal			
	Fodder			
	Aromatic and spicy			
	Oleaginous			
	Melliferous			
	Tinctorial			
	Forestry and anti-erosion			
Vector of introduction	Water			
	Wind			
	Animals			
	Humans			
	Traffic			

* EICAT = Environmental Impact Classification for Alien Taxa, a global standard of the IUCN (International Union for Conservation of Nature) for measuring the severity of environmental impacts caused by invasive alien species.

## Data Availability

The original contributions presented in the study are included in the article. Further inquiries can be directed to the corresponding author.

## Abbreviations

The following abbreviations are used in this manuscript:

ABTS	2,2′-Azinobis-(3-Ethylbenzothiazoline-6-Sulfonic Acid Assay
ATCC	The American Type Culture Collection
BHT	Butylated hydroxytoluene
BDNF	Brain-derived neurotrophic factor
CABI	Centre for Agriculture and Bioscience International
CBD	Convention on Biological Diversity
COX	Cyclooxygenase
DAISIE	Dataset/Inventory of Alien Invasive Species in Europe
DNA	Desoxyribonucleic acid
DPPH	The 2,2-Diphenyl-1-picrylhydrazyl assay
EC	European Commission
EICAT	Environmental Impact Classification for Alien Taxa
EPPO	European and Mediterranean Plant Protection Organization
EU	European Union
FPI	Food Plants International
FRAP	The ferric reducing antioxidant ability
GBIF	Global Biodiversity Information Facility
GISD	Global Invasive Species Database
GISP	Global Invasive Species Program
HGF	Human gingival fibroblasts
IAPS	Invasive alien plant species
INCHEM	International Program on Chemical Safety (IPCS) of the WHO
IPCS	International Program on Chemical Safety
IPBES	Intergovernmental Science-Policy Platform on Biodiversity and Ecosystem Services
ISC	Invasive Species Compendium
ISSG	Invasive Species Specialist Group
IUCN	International Union for Conservation of Nature
MIC	Minimum inhibitory concentration
MMP-9	Matrix metalloproteinase-9
MAO-A	Monoamine oxidase A
MTT	The 3-(4,5-dimethylthiazol-2-yl)-2,5-diphenyl-2H-tetrazolium bromide assay
TEAC	The Trolox Equivalent Antioxidant Capacity
TIMP-1	Tissue inhibitor of metalloproteinases-1
TNF	Tumor necrosis factor
TYR	Tyrosinase
USDA	United States Department of Agriculture
WHO	World Health Organization
WSSA	Weed Science Society of America
